# Identification of ERAP1 protein allotypes in the Turkish population and evaluation of their contributions to Behçet's disease risk

**DOI:** 10.1186/1546-0096-13-S1-P14

**Published:** 2015-09-28

**Authors:** EF Remmers, M Takeuchi, MJ Ombrello, Y Kirino, B Erer, I Tugal-Tutkun, E Seyahi, Y Ozyazgan, A Gul, DL Kastner

**Affiliations:** 1 NHGRI, Inflammatory Disease Section, Bethesda, MD, USA; 2 NIAMS, Translational Genetics and Genomics Unit, Bethesda, MD, USA; 3 Yokohama City University Graduate School of Med, Yokohama, Japan; 4 Istanbul University, Istanbul Faculty of Med, Istanbul, Turkey; 5 Istanbul University, Cerrahpasa Faculty of Med, Istanbul, Turkey

## Introduction

Ankylosing spondylitis, psoriasis, and Behçet's disease are seronegative genetically complex diseases that are also considered complex autoinflammatory diseases. These diseases are characterized by strong association of class I human leukocyte antigen (HLA) alleles and also have a strong contribution to disease-risk by variants of the endoplasmic reticulum-associated amino-peptidase 1 (*ERAP1*) gene that is limited to individuals who carry the disease-specific HLA class I allele. The ERAP1 protein is responsible for trimming peptides for loading onto HLA class I molecules, which are displayed on the surface of nearly all cells, where they play important roles in immune surveillance and in innate and adaptive immune functions. *ERAP1* is highly polymorphic with several SNPs encoding variant amino acids that are likely to influence the nature of peptides bound as well as their ability to be trimmed. These non-synonymous coding variants are not found in isolation, but in combinations or allotypes that act in concert to influence the peptidome available for HLA binding and presentation.

## Objective

To determine the common protein allotypes of ERAP1 in the Turkish population and evaluate their contributions to risk of Behçet's disease.

## Materials and methods

Dense genotyping of the *ERAP1* gene region was performed in 1900 patients with Behçet's disease and 1799 healthy controls from previously reported Turkish GWAS and replication studies using the Immunochip. Additional marker genotypes were imputed. Haplotypes of non-synonymous coding SNPs were determined with SVS (Golden Helix) and disease association was evaluated by comparison of haplotype frequencies in cases and controls.

## Results

SNPs encoding ten amino acid variants of the ERAP1 protein were found with minor allele frequency greater than 1% in 1000 Genomes EUR super-population (ancestral amino acid, position, derived amino acid: T12I, E56K, P127R, I276M, G346D, M349V, K528R, D575N, R725Q, Q730E). These SNPs defined 8 haplotypes with frequency from 2.4 to 23.7%. A single haplotype (0.17 frequency) bearing 5 non-ancestral amino acids (V349, R528, N575, Q725, and E730) was associated with Behçet's disease susceptibility (recessive model, OR = 2.55, 95% CI = 1.70-3.82, P=3.13 x 10^-6^).

## Conclusion

The ERAP1 allotype, T12, E56, P127, I276, G346, V349, R528, N575, Q725, E730, was associated with Behçet's disease. In previously reported studies this ERAP1 variant has been associated with poor peptide trimming. This study supports the hypothesis that a functionally distinct coding allotype of the ERAP1 protein contributes to Behçet's disease risk by altering the production and/or destruction of peptides available for binding class I HLA.

